# Perforated Jejunal Diverticula Secondary to a Large Faecolith: A Rare Cause of the Acute Abdomen

**DOI:** 10.1155/2014/103943

**Published:** 2014-12-30

**Authors:** Peter John Webster, Abigail Hyland, Amarvir Bilkhu, Satheesh Hanavadi, Narinder Sharma

**Affiliations:** Department of General Surgery, Huddersfield Royal Infirmary, Huddersfield, West Yorkshire HD3 3EA, UK

## Abstract

Jejunal diverticula are uncommon and usually asymptomatic. Very rarely, they can lead to acute complications such as bleeding, obstruction, and perforation. This report describes our experience of a case of jejunal diverticula perforation secondary to a large faecolith, with particular focus on the aetiology and management of this rare condition.

## 1. Introduction

Jejunal diverticula have a reported incidence between 0.3% and 2.3% based on a combination of postmortem studies and radiological investigations [1]. Whilst most cases remain asymptomatic, patients may develop complications including chronic abdominal pain, diverticulitis, haemorrhage, obstruction, and in rare cases perforation. In this report, we describe the progress of a 54-year-old male who presented with generalised abdominal pain who subsequently went on to have a laparotomy where a diagnosis of jejunal diverticula perforation secondary to a large faecolith was made.

## 2. Presentation of Case

A 54-year-old man was referred to our unit with a 5-day history of intermittent and sharp abdominal pain, diarrhoea, and vomiting. His past medical history included type 2 diabetes mellitus (tablet controlled), hypertension, hypercholesterolaemia, and obesity. Routine observations showed a systemic inflammatory response and physical examination revealed generalised abdominal guarding. Blood tests showed an acute kidney injury but no hyperleukocytosis. Chest X-ray showed free air under the diaphragm and peritonitis due to hollow viscus perforation was suspected. After resuscitation, the patient was taken to theatre.

**Figure 1:**
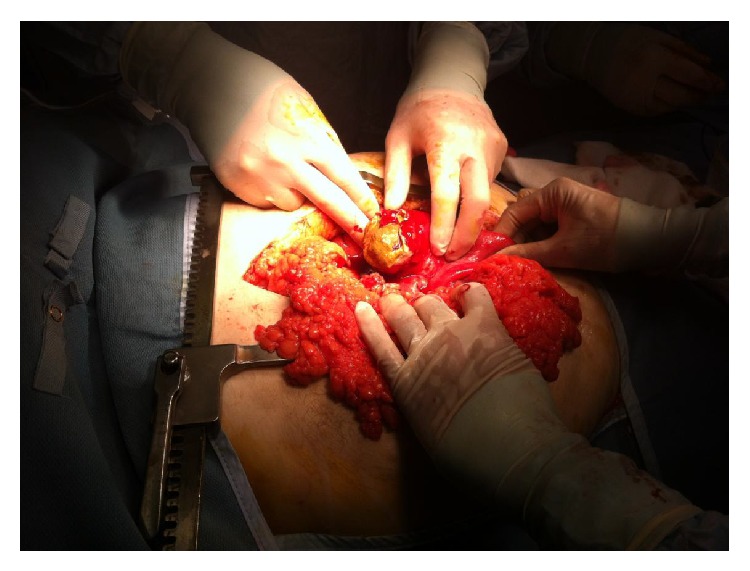
Intraoperative findings: perforated jejunal diverticula with impacted faecolith.

An emergency laparotomy was performed, confirming the diagnosis of generalised purulent peritonitis due to the perforation of a large jejunal diverticula containing an impacted faecolith, 20 cm distal to the duodenojejunal flexure (Figure 1). After resection and peritoneal washing, the intestinal continuity was reestablished with a single layer end-to-end anastomosis. The patient was then transferred to the intensive care unit where he slowly recovered despite a postextubation respiratory failure and the need for vasopressor support. He was discharged home on the 14th postoperative day.

## 3. Discussion

Small bowel diverticulosis was first described by Sommering in 1794 [2] and is thought to result from a combination of intestinal dyskinesia and abnormal peristalsis causing high segmental intraluminal pressures [3]. This results in herniation of the mucosa and submucosa on the mesenteric border at points of weakness where blood vessels enter the bowel wall. In the small bowel, duodenal diverticula are five times more common than jejunal diverticula; however, the complication rate is five times higher in the latter [4, 5].

Jejunal diverticula are more common in males and with age. They are most often asymptomatic and tend to be diagnosed once a complication has occurred such as obstruction, haemorrhage, or perforation [6–8]. In the acute setting, chest and abdominal X-ray may show evidence of perforation or obstruction. CT scan may also identify evidence of complications such as obstruction, perforation, or abscess formation [9]. Even so, the final diagnosis is often only made at the time of surgery. In this case, the patient was relatively young but septic with evidence of viscus perforation on his chest X-ray. Therefore, a laparotomy was carried out without a preoperative CT scan and the diagnosis was made at the time of operating.

Faecolith formation in a jejunal diverticulum is a rare event. Although not fully understood, they are thought to form de novo from choleic acid, the end product of bile salt metabolism [10]. Bacteria within the diverticula deconjugate bile salts, which, in the presence of the acidic pH of the proximal small bowel, results in precipitation and stone formation [10]. This is compounded by stagnation as a result of abnormal transit [3]. Faecoliths can remain in the diverticula and lead to complications such as small bowel obstruction [6–8], and very rarely, perforation from pressure necrosis, or acute necrotizing inflammation [10–12].

Nonsurgical management has been described in stable patients with jejunal diverticular perforations and localised peritonitis [13, 14]. As jejunal diverticula occur at the mesenteric border of the bowel, the small bowel mesentery is available to wall off perforations causing localised peritonitis only. Intravenous antibiotics and CT-guided aspiration of collections may be appropriate and negate the need for surgery in clinically stable patients [13]. However, patients that present with generalised peritonitis or septic shock are best managed surgically with prompt laparotomy, segmental intestinal resection, and primary anastomosis, usually the operation of choice [10–12, 15].

Mortality is influenced by patient's age, comorbidities, and timeliness of intervention. In patients presenting with severe haemodynamic instability, an alternative, safer approach would be resection and exteriorisation of the bowel ends with delayed reestablishment of intestinal continuity. In this case, although the patient had purulent peritonitis with an oedematous bowel, considering the age of the patient and the vascularity of the jejunum, primary resection and anastomosis were carried out.

## 4. Conclusion

Jejunal diverticula are the rarest form of small bowel diverticula and are usually asymptomatic. Perforation of a jejunal diverticulum secondary to a faecolith leading to generalised peritonitis is very uncommon. Evidence exists for nonsurgical management of jejunal diverticula perforations in cases of localised peritonitis in a clinically stable patient, but, in cases of generalised peritonitis, resection of the affected segment with primary anastomosis should be the treatment of choice.
